# Differential regulation of heparan sulfate biosynthesis in fibroblasts cocultured with normal vs. cancerous prostate cells

**DOI:** 10.3389/fimmu.2024.1440623

**Published:** 2024-09-09

**Authors:** Elvira V. Grigorieva, Anastasia V. Strokotova, Ingemar Ernberg, Vladimir I. Kashuba

**Affiliations:** Department of Microbiology, Tumor and Cell Biology (MTC), Karolinska Institute, Stockholm, Sweden

**Keywords:** glycosylation, heparan sulfate biosynthesis, gene expression, transcription factor, transcriptional regulation, prostate cancer cells, fibroblast

## Abstract

Heparan sulfate proteoglycans (HSPGs) regulate a wide range of biological activities in both physiological and pathological conditions. Altered expression or deregulated function of HSPGs and their heparan sulfate (HS) chains significantly contribute to carcinogenesis as well and crucially depends on the functioning of the complex system of HS biosynthetic/modifying enzymes termed as “GAGosome”. Here, we aimed at investigating the expression profile of the system in a cell culture model of stroma-epithelial crosstalk and searching for transcription factors potentially related to the regulation of expression of the genes involved. Coculture of BjTERT-fibroblasts with normal PNT2 human prostate epithelial cells resulted in significant downregulation (2-4-fold) of transcriptional activity of HS metabolism-involved genes (*EXT1/2, NDST1/2, GLCE, HS2ST1, HS3ST1/2, HS6ST1/2, SULF1/2, HPSE*) in both cell types, whereas coculture with prostate cancer cells (LNCaP, PC3, DU145) demonstrated no significant interchanges. Human Transcription Factor RT^2^ Profiler PCR array and manual RT-PCR verification supposed FOS, MYC, E2F, SRF, NR3C1 as potential candidates for regulation and/or coordination of HS biosynthesis. Taken together, transcriptional activity of HS biosynthetic system in normal fibroblasts and prostate epithelial cells during their coculture might be controlled by their intercellular communication, reflecting of adaptation of these cells to each other. The regulation is attenuated or abrogated if normal fibroblasts interact with prostate cancer cells making the cancer cells independent of the limiting effects of fibroblasts, thus contributing to possibility of unlimited growth and progression. Overall, these data demonstrate an ability of cell-cell interactions to affect transcriptional activity of HS biosynthesis-involved genes.

## 1 Introduction

Heparan sulfate proteoglycans (HSPGs) are key components of cell surface and basal membrane in all tissues. HSPGs are actively involved in mammalian development and physiology being involved in cell-cell and cell-matrix interaction and signaling, cell adhesion and migration and numerous signaling pathways (1, 2). In carcinogenesis, HSPGs modulate the tumor microenvironment (3), promoting or inhibiting tumor growth and metastasis depending on their structure and expression levels (4). Furthermore, HSPGs are valuable as tumor biomarkers, aiding in the diagnosis and prognosis of various cancers and can be used as tumor biomarkers and pharmacological targets for anticancer therapy (5, 6).

Polysaccharide HS chains significantly contribute to the functional activities of HSPGs due to their negative charge and highly heterogeneous structure underlying the ability of HSPGs to interact with multiple ligands like growth factors, chemokines and extracellular matrix (ECM) components (7–10) and these interactions are governed by specific sulfation codes of HSPGs (11). HS is actively involved in development (12), neural differentiation (13), immune response and inflammation (14), viral entry (15, 16) as well as in carcinogenesis (4).

HS is one of the most information richest biopolymers in nature (17). The variety is created by a non-template mechanism based on the system of Golgi-located enzymes responsible for HS chain polymerization and post-synthetic modification (18–20). Genes coding these enzymes (*EXT1, EXT2, NDST1, NDST2, GLCE, HS2ST1, HS3ST1, HS3ST2, HS6ST1. HS6ST2, SULF1, SULF2, HPSE*) can be denoted as “HS metabolism-related genes”. Most of them are critically important for life and expression defects are either lethal or result in multiple pathologies (21) as shown in knockout models established for many of the key enzymes involved in HS biosynthesis and modification - EXT1 (22, 23), NDST1 (24–26), GLCE (27, 28), HS6ST1 (29, 30), SULF1 and SULF2 (19, 31).

An important feature of the enzymatic system for HS biosynthesis and modification is its functional unity. All the genes must be appropriately expressed at normal physiological conditions, while omitting of one of them will drastically affect the structure of HS chains or their existence (32). For example, lack of EXT1/2/EXTL gene activities responsible for HS chain elongation will result in a complete HS-null phenotype despite of the expression and full functional activity of all the other HS modifying enzymes. At the same time, lack of any of the HS modifying enzymes will affect the structure and/or overall charge of the synthesized HS chains and their functional activity. So, proper functioning and tight coordination of HS biosynthetic/modifying enzymes is vital for normal physiology (33).

The current data state that the complex of HS biosynthetic enzymes exists as a functional unit called GAGosome (34, 35) or heparanosome (36), where individual enzymes are able to regulate expression/content/enzymatic activity of other team members. It is shown that exostosin glycosyltransferases 1 and 2 (EXT1 and EXT2) responsible for elongation of HS chain during biosynthesis affect expression of N-deacetylase/N-sulfotransferase 1 (NDST1) and HS sulfation (34), decreased expression level of NDST1 results in a higher sulfate content of the synthesized heparin in mast cells (35), although Ndst2 does not affect Ext1 and Ext2 expression in Ndst2(-/-) mice (37). Additional Hsepi and Hs6st interactions in heparan sulfate biosynthesis have been predicted by computational modeling (38). According to other data, an alternative model for the GAGosome can be proposed, in which HS itself may induce the formation of enzymes complexes that transiently put together around newly synthesized polysaccharide chains (39).

At the transcriptional level, each investigated tissue *in vivo* is characterized by tissue-specific transcriptional patterns and expression levels of HS biosynthetic system, although cell type-dependent variation were observed in different cell lines of the same origin *in vitro* (40). Specific changes in expression of HS metabolism-involved genes and expression patterns of HS metabolic system occur in different cancers (40) - expression of nine HS-modifying enzymes (especially those involved in HS sulfation) are significantly changed in hepatocellular cancer (41); breast tumors are characterized by the altered expression of 3-O-sulfotransferase, NDST4, sufatases and heparanase-2 resulting in cancer-specific patterns of HS biosynthesis (42); overall impairment of transcriptional activity of HS metabolic system and changes in the expression patterns of HS metabolism-involved genes have been shown in glioblastoma (43) and prostate tumors (44). Besides that, the transcriptional activity of the HS metabolism-involved genes responds to different drugs such as dexamethasone or/and temozolomide which affect that in both normal murine brain tissue (45) and U87 xenografts grown in SCID mice (46). All the data demonstrate responsiveness of the HS biosynthetic system to different stimuli/pathological conditions and suppose deterioration of a regulating mechanism for the coordinated transcription of HS metabolism-involved genes in such conditions.

Earlier, we established an experimental system *in vitro* to study an ability of the interacting cells (fibroblasts, FB and prostate epithelial cells) to affect their molecular parameters related to cell-cell communication. It was shown that coculture of these cells results in the cell type-specific changes in morphology and proliferation rates of the interacting cells, and there are evident differences in the interaction mode of FB with normal and cancer prostate epithelial cells in terms of the expression levels of HSPG core proteins and a number of cell adhesion molecules (47). However, an ability of interacting cells to modify/regulate transcriptional activity of the genes responsible for biosynthesis of polysaccharide HS chains remained uninvestigated. In this study, we aimed to investigate differential expression of HS metabolism-related genes in human FBs and prostate epithelial cells in the cell coculture model *in vitro* as well as the expression of different transcription factors (TFs) to reveal candidate TFs potentially involved in the transcriptional regulation and coordination of HS biosynthetic system.

## 2 Materials and methods

### 2.1 Cell lines and coculture assay

Cell were obtained and cultured as it is presented in (47). Briefly, the human TERT-immortalized fibroblasts (BjTERT) and prostate cancer cell lines LNCaP, PC3 and DU145 were obtained from MTC (Karolinska Institute, Stockholm, Sweden). The normal human prostate epithelial cell line PNT2 was obtained from the European Collection of Cell Cultures (ECACC, Salisbury, UK). All cell lines were maintained in RPMI medium supplemented with 2 mM L-glutamine, 100 units penicillin, 100 µg/ml streptomycin, and 10% (v/v) fetal bovine serum at 37°C in a humidified 5% CO_2_ incubator. Cells were harvested for analysis using trypsin/EDTA. For coculture experiments, fibroblasts and prostate epithelial cells were seeded at a 1:1 ratio with 25-30% confluency at the starting point, and the cells were then separated after 72h of incubation as described below.

### 2.2 Magnetic separation of different cell types upon coculture

After coculture, BjTERT fibroblasts and epithelial cells were separated based on Miltenyi Biotec’s MAgnetic Cell Separation (MACS) technology (Miltenyi Biotec, Germany) according to the manufacturer’s instructions. Briefly, the cell suspension was mixed with magnetic Anti-Fibroblast MicroBeads conjugated to monoclonal mouse anti-fibroblast human antibodies (cat. N 130-050-601) and incubated for 30 minutes at room temperature. The cell suspension was applied onto a MACS column (featuring a cell-friendly matrix) placed into an OctoMACS separator equipped with powerful magnets (which amplifies the magnetic field within the matrix). This field efficiently captures magnetic-labeled cells while ensuring the gentle flow of unlabeled prostate epithelial cells through the column. The flow-through fraction, containing unlabeled, was collected. Fibroblasts labeled with magnetic beads were then eluted from the MACS column using elution buffer by remove the column from the magnetic field. This enables the elution process to enrich for our positively labeled cells. Collected labeled (fibroblasts) and unlabeled (prostate epithelial cells) fractions were used in further experiments.

### 2.3 RNA isolation and real-time RT-PCR

RT-PCR analysis was performed as it is presented in (47). Briefly, Total RNA was extracted from the cells using the RNeasy Plus Mini Kit (Qiagen, USA) according to the manufacturer’s instructions. Total RNA concentration was measured withQubit–iT RNA Assays Kit (ThermoFisher Scientific, USA) according to the manufacturer’s instructions. cDNA was synthesized from 1–2 µg of total RNA using a RevertAid First Strand cDNA Synthesis Kit (Fermentas, USA), and 1/10th of the product was subjected to PCR analysis.

Real-time PCR was performed using Maxima SYBR Green/ROX qPCR reagent (ThermoFisher Scientific, USA) on Applied Biosystems™ 7500 Real-Time PCR system under the following conditions: 95°C for 10min, 95°C for 20sec, 60°C for 20sec and 72°C for 50 sec. The total reaction volume was 25*μ*l. The RT-PCR results were quantified using the 2^-ddCt^ method and were normalized against *GAPDH*. The human PCR primers used are present in (43).

### 2.4 Human Transcription Factor RT^2^ Profiler PCR array

The Human Transcription FactorRT^2^ Profiler PCR array (PAHS-075Z, SABioscience, USA) was used to determine changes in the expression of 84 different transcription factors in fibroblasts before and after their coculture with normal or prostate epithelial cells. Briefly, total RNA was isolated using aRNeasy Plus Mini Kit (Qiagen, USA). The RNA concentration was determined using a Quant-iT Assay Kit for RNA quantification (ThermoFisher Scientific, USA). cDNA was synthesized from 1–2*μ*g of total RNA using a Maxima First Strand cDNA Synthesis Kit for RT-qPCR (ThermoFisher Scientific, USA). Real-Time PCR was performed using an RT^2^ Profiler PCR Array Human Transcription Factors with SYBR Green Fluor q-PCR Master Mix (Qiagen, USA) and Applied Biosystems™7500 Real-Time PCR system according to the manufacturer’s instructions. All data were analyzed using Excel-based RT^2^Profiler PCR Array Data Analysis Software v3.5 (SABioscience, USA). This integrated web-based software package automatically calculates ddCt-based fold changes in gene expression from the uploaded raw threshold cycle data. Each replicate cycle threshold (Ct) was normalized by the software to the RPLP0 gene Ct on a per plate basis.

### 2.5 Statistical analysis

Statistical analyses were performed using a computer program ORIGIN Pro 8.0; a value of p<0.05 was considered to indicate a statistically significant difference. Data are expressed as the means ± SEM. Spearman Rank Order Correlations coefficients were determined to analyze the correlation between the studied genes.

## 3 Results

### 3.1 Transcriptional activity of HS metabolic system in fibroblasts and prostate epithelial cells upon coculture *in vitro*

To explore an involvement of the HS metabolic machinery in cell-cell communication between BjTERT-immortalised human fibroblasts (FB) and normal (PNT2) or morphologically different cancer prostate epithelial cells (LNCaP, PC3, DU145). These cell lines possess different tumorigenic properties (hormone-dependent non-metastatic LNCaP cells and hormone-independent metastatic PC3 and DU145 cells) potentially affecting their interaction with fibroblasts. Comparative analysis of that for normal (PNT2) and prostate cancer cells is appropriate cell model for elucidating of involvement of HS into intercommunication of different cell types.

**Figure 1:**
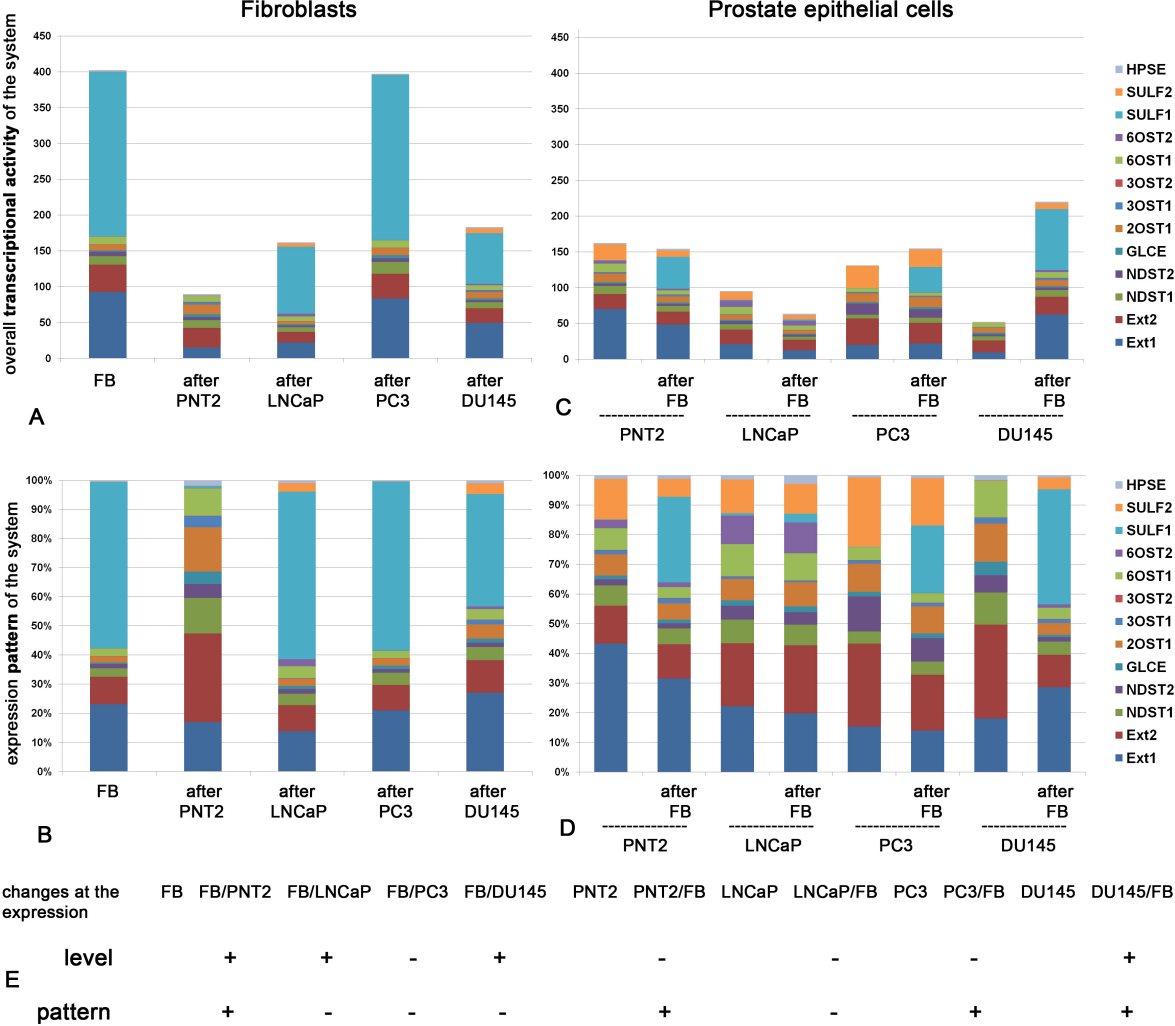
Transcriptional activity of HS metabolic system in fibroblasts and normal or cancer prostate cells alone and after coculture. Overall expression level of the genes **(A, C)** and their expression patterns **(B, D)** in fibroblasts **(A, B)** and epithelial prostate cells **(C, D)**. The stacked columns reflect the contribution of each gene to the total expression level. Real-time RT-PCR analysis. Intensity of the amplified DNA fragments normalized to that of *GAPDH*. **(E)** Schematic overview of the quantitative and qualitative changes in HS metabolic system in fibroblasts (FB) and normal (PNT2) or cancer (LNCaP, PC3, DU145) prostate cells before and after coculture. (+) - the parameter is changed after coculture (–), - no change after coculture.

These cells were cocultured *in vitro* as previously reported (47) followed by analysis of the expression pattern and transcriptional activity of HS metabolism-involved genes (**Figure 1**). Expression level represents quantitative parameter reflecting overall transcriptional activity of the studied cells in terms of the expression of HS biosynthesis-involved genes (**Figures 1A, C**), whereas expression pattern is a qualitative parameter reflecting the ratio of expression levels of these genes presented as a percentage of each gene of the total 100% (**Figures 1B, D**).

**Figure 1** shows transcriptional activity of HS metabolic system in fibroblasts (**Figures 1A, B**) and normal or cancer prostate cells (**Figures 1C, D**) alone and after coculture.

In panels A, we observe that overall transcriptional activity of HS biosynthetic system in fibroblasts is significantly decreased upon their coculture with normal PNT2 cells. Statistical analysis demonstrate that the decrease is due to mainly EXT1 and SULF1 down-regulation (ANOVA, p<0.05). Cancer cells affected this parameter cell-line specifically (if any). LNCaP and DU145 decreased the overall transcriptional activity (due to EXT1/2, SULF1 downregulation, p<0.05) but PC3 cells did not affect the studied parameter. Along with these changes in overall transcriptional activity of HS biosynthetic system, coculture with normal PNT2 cells resulted in significant qualitative changes in the expression pattern of this system in fibroblasts (**Figure 1B**), whereas cancer cells failed to affect the expression patterns of HS metabolism-involved genes (in spite of the decrease of overall expression levels of some genes).

In contrast, panels C and D demonstrate changes, which occur in normal (PNT2) and cancer prostate epithelial cells (LNCaP, PC3, DU145) upon their coculture with fibroblasts. Surprisingly, we did not detected significant quantitative changes in the overall transcriptional activity (except DU145 which demonstrated even increase of the parameter due to EXT1 and SULF1 upregulation (p<0.05) (**Figure 1C**). However, the expression patterns of the system in the epithelial cells were modified upon coculture of the cells with fibroblasts in cell type-specific manner (**Figure 1D**). The less aggressive cancer LNCaP cell line did not react to the coculture, whereas expression patterns of HS biosynthetic system in more aggressive hormone-independent PC3 and DU145 cells were modified upon the coculture. Interesting, the expression pattern of the most aggressive DU145 cells became very similar to that in normal PNT2 cells. This similarity might reflect an ability of DU145 cells to synthesize “close-to-normal” HS suggesting potential “mimicry” of DU145 cells (on this parameter) as normal ones to escape immune control and survive.

According to the obtained results, HS biosynthetic system in BjTERT fibroblasts was quantitavely (**Figures 1A, C**) and qualitatively (**Figures 1B, D**) affected upon coculture with normal prostate PNT2 cells *in vitro*. Interesting, co-culture with fibroblasts drastically changed overall transcriptional level of HS-biosynthetic genes in DU145 cells only and expression patterns in PNT2, PC3 and DU145 cells (**Figure 1D**), while LNCaP prostate cancer cells did not respond to the fibroblasts in terms of HS metabolic machinery.

Totally, co-culture of normal fibroblasts and prostate PNT2 epithelial cells resulted to the significant changes in the overall transcriptional activity and/or expression pattern of HS metabolism-related genes in both normal cell lines (FB and PNT2), which may reflect the mutual adaptation of these cells. To note, not all genes showed changes in their expression level after coculture, and this is what led to a qualitative changes in the expression pattern. The contribution of each individual gene into the expression pattern of the system was different, and this in turn depended on the basic level of its expression.

Prostate cancer cells affected fibroblasts and respond to them in terms of transcriptional activity or pattern of HS metabolic system in a very specific and restricted way, demonstrating an autonomic behavior in this regard.

The molecular mechanisms of the inhibition of transcriptional activity of the HS metabolic system in normal FB cells upon coculture with PNT2 cells are unclear. TFs are among the first candidates possibly capable of regulating expression of HS metabolism-involved genes at the transcriptional level.

### 3.2 Human Transcription Factors RT^2^ Profiler PCR array analysis

**Figure 2:**
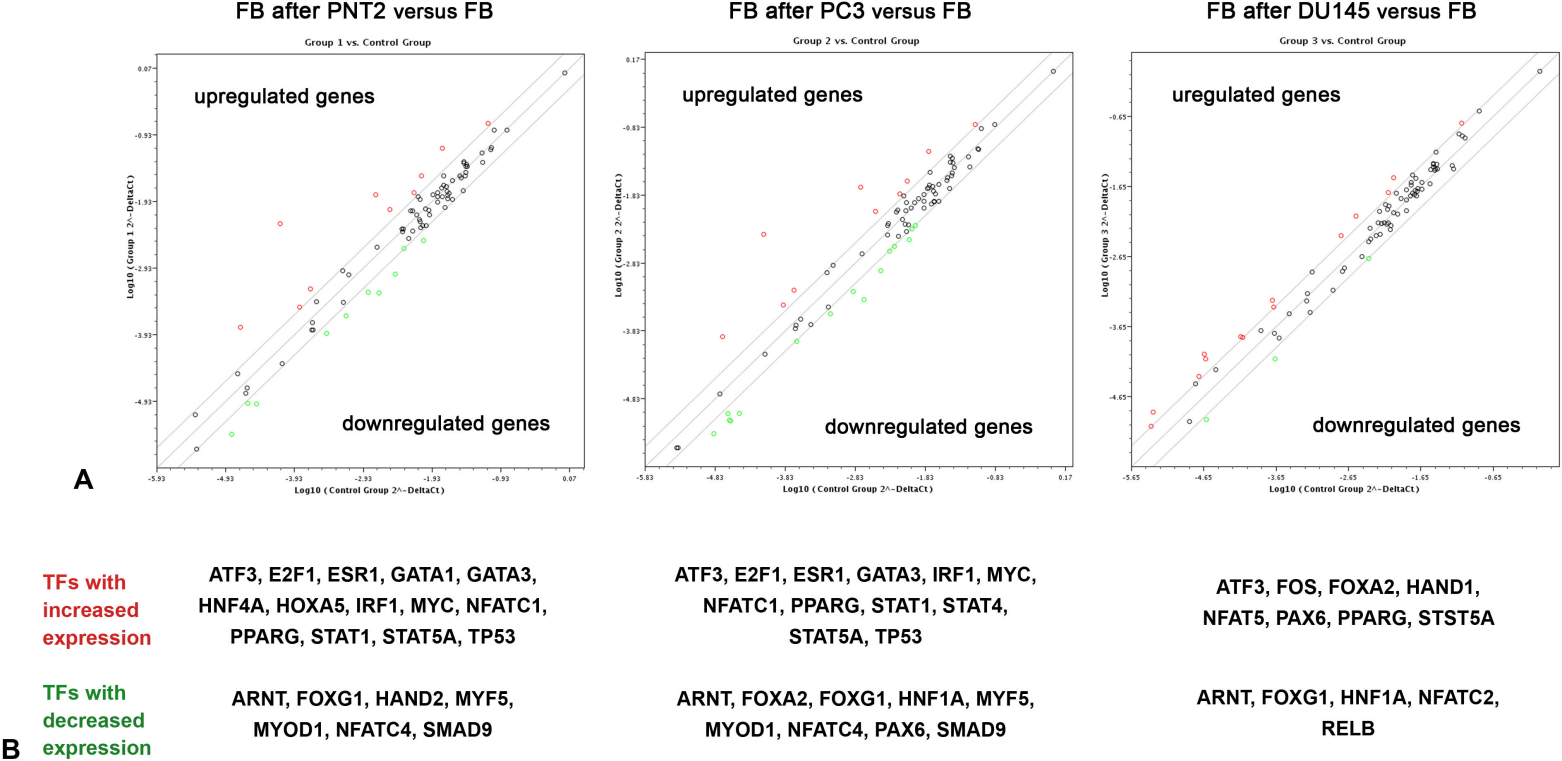
RT^2^ Profiler™ Human Transcription Factors PCR Array analysis of BJhTERT fibroblasts before and after coculture with normal and cancer prostate epithelial cells. **(A)** The relative expression levels for each gene in fibroblasts after co-culture with PNT2 (Group 1), PC3 (Group 2) or DU145 (Group 3) cells are plotted against the same genes from the control fibroblasts (Control Group). The middle line shows the similar expression in both groups with 2-fold change boundaries. Genes up-regulated>2-fold lie above the middle line (red color) and the down-regulated genes lie below the line (green color) (RT^2^ Profiler™ PCR Array Data Analysis software version 3.5). **(B)** Genes, which were up- or down-regulated in the cells after coculture, divided by the normalized gene expression in the control cells before. 2-fold change was considered significant.

To look for potential transcriptional regulators of the HS biosynthetic system, commercial Human Transcription Factors RT^2^ Profiler PCR array (SABioscience, USA), which designed to profile expression of 84 different transcription factors in the target samples, was used. Because expression of HS biosynthesis-involved genes in normal FB and PNT2 cells is evidently attenuated by their coculture, the expression levels of TFs from the Array were determined in FB cocultured with PNT2, PC3 and DU145 cells, representing a model of coordinated deregulation of the transcriptional activity of HS metabolism-involved genes (**Figure 2**).

**Figure 2** shows expression levels of 84 TFs which are marked as dots on the three individual graphs (fibroblasts after coculture with PNT2, PC3 and DU145 cells). On these logarithmic graphs, the middle line denotes no changes in the expression level of each gene in cocultured fibroblasts (experimental group) compare with intact fibroblasts (control group), while two boundary lines denote 2-fold change (up-regulation or down-regulation) in the expression. Up-regulated and down-regulated genes are marked with red and green color (respectively).

In panel A we observe that relatively few TFs were affected in fibroblasts upon their coculture with prostate epithelial cells (both normal and cancer) - 21 of 84 genes for PNT2, 21 of 84 genes for PC3, 13 of 84 genes for DU145 (**Figure 2A**). All the genes (TFs) with the more than 2-fold changes in the expression level in fibroblasts co-cultured with PNT2, PC3 or DU145 are shown on panel B (**Figure 2B**). Some of the TFs (ATF3, PPARG, ARNT, FOXG1) seem to be common in reacting fibroblast to the presence of any cell type (PNT2, PC3 or DU145), whereas other demonstrate cell type-dependence. In this term, PNT2 and PC3 affect fibroblasts in more similar way activating expression of E2F1, ESR1, GATA3IRF1, MYC, NFATC1, STAT1, TP53, while coculture with DU145 activate expression FOS instead (**Figure 2B**).

This approach let identifying sets of TFs which expression level in fibroblasts depends on the culture mode of these cells – either monoculture or coculture with prostate epithelial cells.

### 3.3 Real-time RT-PCR analysis

**Figure 3:**
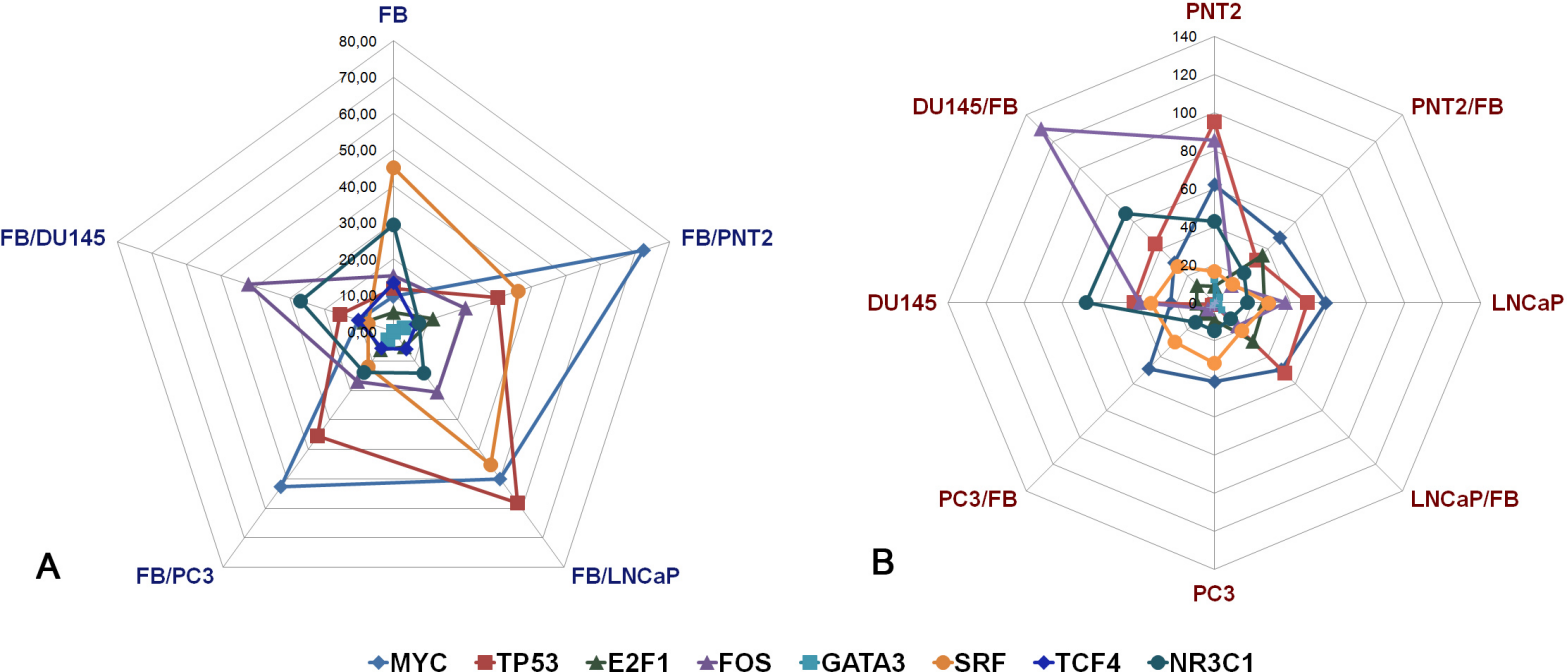
RT-PCR analysis of the expression levels of selected TFs in fibroblasts **(A)** and normal or cancer prostate cells **(B)** before and after their coculture. Intensity of the amplified DNA fragments normalized to that of *GAPDH*. FB - fibroblasts, PNT2 - normal prostate epithelial cells, LNCaP, PC3, DU145 - prostate cancer cell lines.

To investigate the matter further, TFs the most affected during coculture of different cell types (E2F1, TCF4, GATA3, MYC, TP53, SRF, FOS, NR3C1) were chosen for manual verification by real-time RT-PCR analysis. Expression levels of the selected TFs were determined in the same fibroblasts and normal or cancer prostate epithelial cells before and after co-culture by real-time RT-PCR analysis (**Figure 3**).

FB in monoculture or after coculture with normal and cancer prostate cells demonstrated heterogeneous changes in the expression of the studied TFs (**Figure 3A**). E2F1, TCF4 and GATA3 had very low expression levels both in intact FB and in FB after co-culture, and there were no significant changes in their expression levels upon coculture. MYC, TP53, SRF, FOS, NR3C1 showed the highest expression levels, and responded to coculture with prostate epithelial cells by the different ways – normal PNT cells significantly upregulated TP53 and MYC (+3-7-fold, p<0.05) and downregulated NR3C1 (-4-5-fold, p<0.05), whereas cancer cells induced heterogeneous changes in TP53, MYC, FOS, SRF, NR3C1 expression (**Figure 3A**).

Normal prostate epithelial PNT2 cells reacted to coculture with FB by decrease of TP53 (-3-fold, p<0.05), FOS (-8-9-fold, p<0.01) and NR3C1 (-2,5-3-fold, p<0.05)) (**Figure 3B**). At the same time, prostate cancer LNCaP and PC3 cells did not respond to FB significantly in terms of TFs expression, with exception of DU145 demonstrating just +3-fold upregulation of FOS (p<0.05).

**Figure 4:**
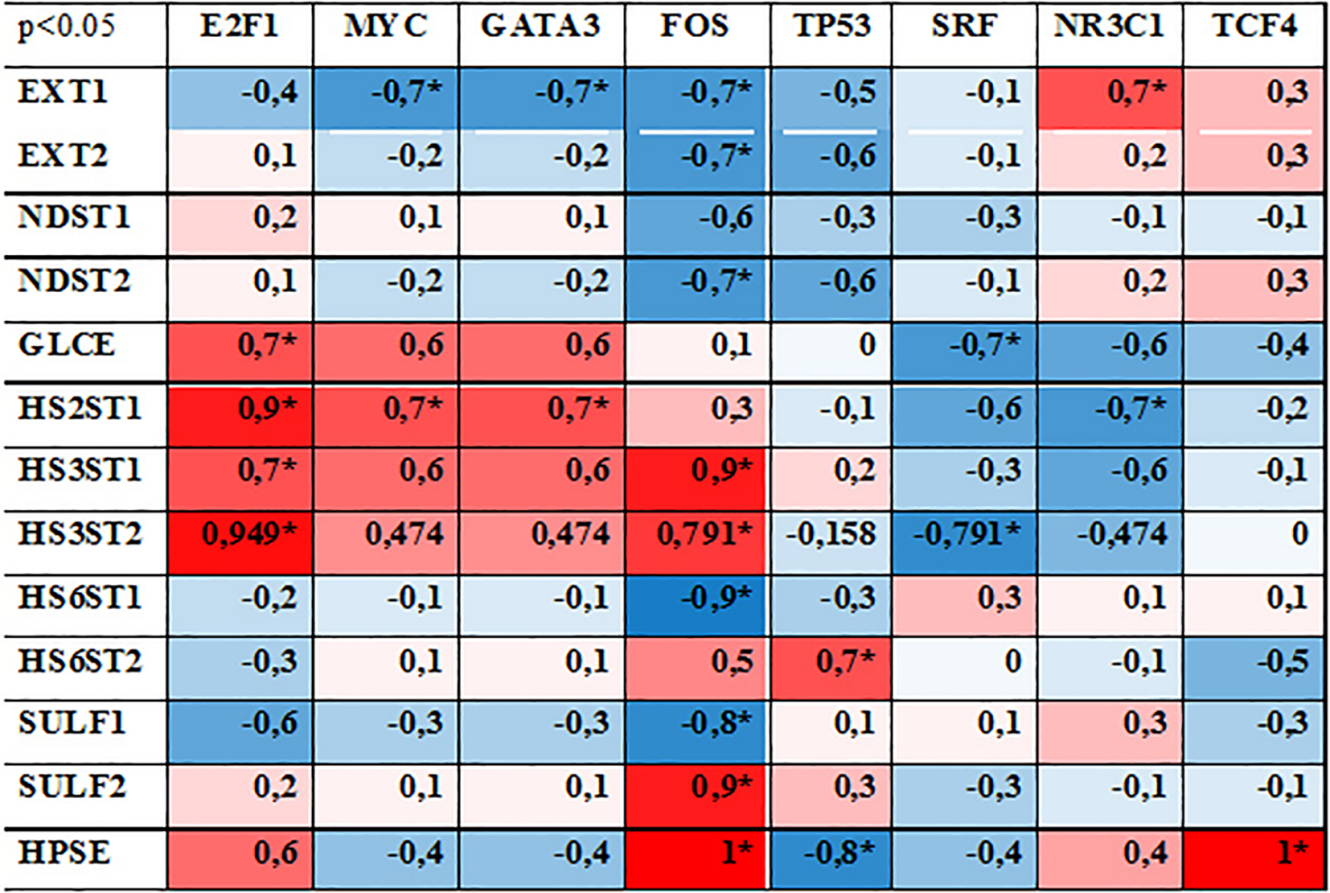
Correlation analysis between the expression levels of studied TFs and HS metabolism-involved genes. Spearman Rank Order Correlations coefficients, MD pairwise deleted. Marked correlations are significant at p <0,05. Blue represents a negative correlation, red represents a positive correlation coefficient. * - significant correlation.

To estimate whether these changes in TFs expression are associated with the transcriptional activity of HS metabolic system, correlation analysis was performed. Expression levels of each TF was correlated with that for each HS biosynthesis-related gene and Spearman Rank Order Correlation coefficients were depicted in the table format (**Figure 4**). Correlation coefficients more 0.7 or lower -0.7 was taken as strong positive or negative correlation (respectively).

According to the original hypothesis, TFs capable to coordinate transcriptional activity of the complex of HS biosynthesis-involved genes should demonstrate simultaneous correlation with the most of the genes. However, none of the studied TFs showed a high correlation (positive or negative) with all HS biosynthesis-involved genes. The most of the studied TFs (MYC, GATA3, TP53, SRF NR3C1, TCF4) demonstrated random correlations with the expression level of 1-2 genes (mainly EXT1, GLCE, HS2ST1, HS3ST1, HS3ST2, HPSE) reflecting a potential of these TFs to regulate these genes’ expression.

Among the studied TFs, FOS expression levels were more tightly correlated with transcriptional activity of a number of HS-related genes (9 of 13 genes, correlation coefficients 0,7-1,0). However, if for HS chain elongation-responsible enzymes (EXT1/2, NDST1/2), O-heparan sulfate 6-sulfotransferase 1 (HS6ST1) and sulfatase 1 (SULF1) there was a negative correlation with FOS expression, expression of O-heparan sulfate 3-sulfotransferases (HS3ST1/2) and sulfatase 2 (SULF2) was positively correlated. One could speculate that it can reflect an existence of separate mechanisms for the transcriptional regulation of some functional “blocks” inside HS biosynthetic system (HS chain elongation, epimerization, 2/3-sulfation, 6-sulfation, desulfation). This hypothesis is indirectly supported by high positive correlation of E2F1 expression with epimerization (GLCE)-2/3-sulfatation (HS2ST1, HS3ST1/2) enzymes only. Similar effect (although milder and not statistically significant) was observed for MYC (+0,47-0,7) and GATA3 (+0,47-0,7), whereas expression of SRF (–0,3–0,79) and NR3C1 (–0,47–0,7) were negatively correlated with these enzymes. At the same time, the expression of these TFs did not correlate with any other enzymes from the HS biosynthetic system.

In summary, interaction of normal cells (FB and PNT2) resulted in the noticeable changes in the TFs expression in both cell types. Cancer cells affected TF expression in FB, while remaining weakly responsive to FB. Among the studied TFs, FOS, MYC, GATA3, SRF and NR3C1 were the most expressed and sensitive to coculture of different cell types and correlated with the expression levels of some HS biosynthesis-involved genes. However, it is important to note that correlation does not imply causation. Therefore, additional studies are necessary to verify these findings and to understand the underlying mechanisms.

## 4 Discussion

Upon the coculture of fibroblasts with normal PNT2 cells, there was an evident reorganization of overall transcriptional activity and expression pattern of HS metabolic system in both the fibroblasts and PNT2 cells. This may reflect a cooperation of the cells in terms of the biosynthesis of certain macromolecules necessary for their joint functioning - some functions are transferred to one cell type, while others are provided by its neighbor. For example, in monoculture, sufatase 1 (SULF1, secreted extracellular endosulfatase which selectively removes 6-O-sulfate groups from HS chains) is highly expressed in FB (**Figure 1A**) but not PNT2 cells (**Figure 1C**). After coculture these cells, fibroblasts lose the ability to express SULF1 (**Figure 1A**), while PNT2 cells acquire it (**Figure 1C**) supposing these cells as a main source of sulfatase 1 in FB-PNT2 coculture. Since this enzyme plays a crucial role in the sulfation of HS (48), such changes may have functional significance for the interaction of HS with its ligands.

In contrast, prostate cancer cells had a quantitative, but not a qualitative effect on the HS metabolic system in FB - LNCaP and DU145 significantly (-2-3 fold) inhibited expression of HS metabolism-involved genes retaining their expression pattern, whereas PC3 cells had no effect at all. These results stay in line with the data on the altered expression of the HSPG biosynthesis-involved genes in breast cancer (42) and gliomas (43) and extend our previously published results for prostate cancer that LNCaP and PC3 cells possess very low or no sensitivity to FB presence in coculture system *in vitro* (44). Moreover, the changes in the HS metabolic machinery coincide with the changes in the expression level and pattern of HSPG core proteins in the same coculture cell model (47) supporting an idea about interrelation of transcriptional activities of HSPG core proteins with the expression of the system responsible for biosynthesis of their HS chains.

The HS biosynthetic/metabolic machinery requires a system for coordinated regulation of the expression of these genes located in different chromosomes but involved in one functional system (44). This presumed regulation would coordinate expression of individual genes converting them into a well-coordinated HS biosynthetic system. Changes in this regulatory mechanism might contribute to deterioration of intercellular interactions between the normal FB and prostate cancer cells. Kreuger and Kjellen (32), which provides the current understanding of HS biosynthesis and its regulation, review regulatory mechanisms of HS biosynthesis for individual HS metabolism-involved genes. These mechanisms might be of a great importance for understanding of the role of HS structure in HS-protein interactions and development of their therapeutic targeting (8). The presented results stay in line and support up-to-date concept on the ability of epithelial and stromal cells to precisely tune the availability of signaling molecules and modulate ligand-receptor interaction and intracellular signal transduction by controlling of HS biosynthesis and sulfation pattern, as well as the cleavage of the HS chain and/or the shedding of proteoglycans (49).

In this study, we have investigated a potential interrelation of the expression levels of various TFs with the expression levels of HS metabolism-involved genes (**Figures 2**, **3**) and identified E2F1, FOS, MYC, TP53, NR3C1 as potentially capable to regulate and/or coordinate transcriptional activity of these genes. It is difficult to compare these results with the literature data, since there is currently very little data on the regulation of HS biosynthesis-involved genes by TFs. It was shown that Runt-related transcription factor 2 (Runx2/Cbfa1) increases expression of Ext1 and heparanase, as well as alters the relative expression of N-linked sulfotransferases (Ndst1 = Ndst2 > Ndst3) and enzymes mediating O-linked sulfation of HS (Hs2st > Hs6st) in osteoprogenitor cells (50). Hypoxia-inducible factor 1-alpha (HIF-1-alpha) (a subunit of a heterodimeric transcription factor HIF–1) capable to increase expression levels of GlcNAcT-I and HS2ST, thus promoting preferential synthesis of HS rather than CS chains and increasing the number of FGF2-binding sites on HS chains (51). Two cis-acting binding elements for the beta-catenin-TCF4 complex are located in the enhancer region of the GLCE promoter, and the ectopic expression of beta-catenin-TCF4 in colon carcinoma cells produces a significant increase of GLCE transcript level and enhances the rate of D-glucuronic acid epimerization in HS, suggesting that the beta-catenin-TCF4 transactivation pathway plays a major role in modulating of GLCE expression (52). The involvement of TCF4/β-catenin in regulating GLCE expression was shown also for MCF7 breast cancer cells both *in vitro* and *in vivo* (53). Genome-wide CRISPR/Cas9 screen to identify novel regulatory factors of HS biosynthesis revealed the alpha globin transcription factor, TFCP2, as a top hit. A375 TFCP2 knockout human melanoma cells have decreased expression of HS3ST3A1 and HS6ST2 and increased expression of SULF1 and SULF2 what allowed the authors to state TFCP2 as a novel transcriptional regulator of HS biosynthesis (54). Interestingly, SULF1 expression is upregulated upon knockdown of the TFCP2 expression in the cells suggesting not direct effect of the TF onto SULF1 expression. As to the TFs identified in our study, the closest results are presented in few recent articles. Chromatin immunoprecipitation sequencing and RNA-sequencing established an inducible PPARγ-p53 transcription factor complex mediated regenerative program regulating 19 genes involved in lung endothelial cell survival, angiogenesis and DNA repair including *SULF2* (55). Glucocorticoid receptor (Nr3c1) mRNA level in normal mouse brain tissue is associated with the expression of 4 of the 13 HS biosynthesis-related genes supposing an involvement of Nr3c1 in regulation of the expression of these genes (56). In summary, the published data on this issue not very abundant and did not allow to draw a certain conclusion on their agreement/disagreement. At the moment, information on this issue is being accumulated and the data presented here contribute to a better understanding on potential involvement of TFs to the molecular mechanisms of regulation of transcriptional activity of HS biosynthetic system.

Of course, we are far from believing that the regulation/coordination of HS biosynthetic system is carried out only by TFs. Almost certainly, a complex mechanism includes many regulators/confounding factors such as microRNA (57–61) or other regulators like oxidative stress (62). They represent very important regulators of the genes expression and detailed information can be found in the mentioned references. Here, we investigated just on one potential molecular mechanism such as an involvement of TFs, and this is an evident limitation of the study. Nevertheless, the obtained results contribute to investigation of HS biosynthesis coordination drawing a potential direction for future research. Altogether, the factors most likely cooperate in the coordination of HS biosynthesis and as long as this coordinating system exists, all external influences will have only a quantitative effect (uniformly reducing or increasing the expression HS biosynthesis-involved genes), whereas for qualitative changes it will be necessary to overcome this coordinating system or destroy it.

Taken together, the data obtained demonstrate that coculture of fibroblasts with normal PNT2 human prostate epithelial cells results to the changes in the transcriptional activity and pattern of HS biosynthetic system in both cell types, and these changes reflect a physiological adaptation of different cell types to mutual existence. During coculture of FB with cancer cells, the absence of changes is a sign of a pathological disturbance of mutual influence of these cells on each other. The changes in the level of the total transcriptional activity of the studied genes while maintaining their expression pattern supports the existence of a molecular mechanism coordinating the expression of individual HS metabolism-involved genes in this system. Transcription factors E2F1, TP53, MYC, FOS, NR3C1, SRF might be potential regulators of coordinated expression of HS biosynthetic system. The results presented here extend our knowledge about the multiple levels of epigenetic regulation of the HS metabolism-involved genes and support an involvement of TFs in the molecular mechanism of the transcriptional regulation of HS biosynthesis-involved genes.

## Data Availability

The original contributions presented in the study are included in the article/supplementary material, further inquiries can be directed to the corresponding author/s.
